# Adverse Endocrine-Related Effects of Pembrolizumab Precipitating Severe Hyponatremia

**DOI:** 10.7759/cureus.28393

**Published:** 2022-08-25

**Authors:** Muaamar B Baldawi, Balreet Dhami, Jiten Gosai, Ranya H Al-Khafaji

**Affiliations:** 1 Department of Internal Medicine, Northeast Arkansas (NEA) Baptist Memorial Hospital, Jonesboro, USA; 2 Department of Nephrology, The University of Texas Health Science Center, Houston, USA; 3 Medicine, Independent Researcher, Jonesboro, USA

**Keywords:** severe hyponatremia, pembrolizumab, hypothyroidism, adrenal insufficiency (ai), immune-checkpoint inhibitors

## Abstract

Pembrolizumab is an immune-checkpoint inhibitor (ICI), designed as a highly selective anti-programmed death-1 (PD-1) humanized monoclonal antibody, which ultimately inhibits the negative immune regulation due to PD-1 receptor signaling. This inhibition allows for anti-tumor response and reverses T cell suppression. We present a case report of a 53-year-old woman who was treated with pembrolizumab for metastatic melanoma. There have been adverse endocrine-related effects of pembrolizumab, as well as other ICI medications, including hypothyroidism and secondary adrenal insufficiency. Adrenal insufficiency and hypothyroidism can precipitate hyponatremia through different mechanisms. We report a case of a patient developing severe hyponatremia likely precipitated by endocrine-related side effects of pembrolizumab.

## Introduction

T cells target tumor cells through two types of signaling; antigen-specific signaling through T cell receptor (TCR) and antigen nonspecific signaling mediated by CD-28, while programmed death-1 (PD-1) and cytotoxic T-lymphocyte-associated protein-4 (CTLA-4) are co-inhibitory receptors [1]. Pembrolizumab is an anti-PD-1 antibody approved for numerous malignancies, including gastric, ovarian, non-small cell lung cancer, and melanoma [2]. Adverse endocrine-related effects of pembrolizumab include hypophysitis, hypothyroidism, diabetes mellitus (either type 1 or type 2), and primary adrenal insufficiency [3,4]. 

Hyponatremia is defined as serum sodium of less than 135 mmol/L and is considered to be primarily a water balance disorder with excess water. Serum osmolality needs to be determined next to classify hyponatremia and to rule out pseudohyponatremia. Pseudohyponatremia is commonly caused by an increased concentration of osmotic molecules, other than sodium, in the serum. Hyperglycemia is a common culprit. Hypoosmolar hyponatremia is further classified into hypovolemic, hypervolemic and euvolemic. Briefly, hypoosmolar hypervolemic hyponatremia is usually precipitated by conditions such as heart failure and cirrhosis. Hypoosmolar euvolemic hyponatremia may be caused by the syndrome of inappropriate antidiuretic hormone (SIADH), glucocorticoid deficiency, and severe hypothyroidism. Hypoosmolar hypovolemic hyponatremia may be caused by adrenal insufficiency, cerebral salt wasting, and diuretic use. Hypoosmolar hypovolemic hyponatremia can be precipitated by renal and extrarenal loss [5,6].

Clinical presentation of hyponatremia is dependent on the severity and the acuity of hyponatremia ranging from nausea, vomiting, confusion, and headaches to somnolence, seizure, and coma. Patients also present with symptoms related to their volume status [7]. Management of hyponatremia consists of correcting the sodium level safely and addressing the underlying etiology [6].

## Case presentation

The patient is a 53-year-old woman with a past medical history significant for metastatic melanoma presented to Northeast Arkansas (NEA) Baptist Memorial Hospital due to poor oral intake for four to five days. The patient's husband was at the bedside providing history. He reported that the patient had nausea, vomiting, and diarrhea for the past two days. She also had shortness of breath for the past three days. 

Vital signs revealed hypotension (75/51 mmHg) with mean arterial pressure (MAP) of 59 mmHg. Physical examination revealed a lethargic, minimally responsive, and cachectic appearing female without evidence of anasarca. Her lungs were clear to auscultate. Her volume status was difficult to assess, but she was likely euvolemic to mild hypovolemia. She was found to have significant electrolytes abnormalities, including hyponatremia at 106 mmol/L, hypocalcemia at 7.0 mg/dL, hypochloremia at 76 mEq/L, hypokalemia at 2.4 mEq/L, and hypophosphatemia at 1.3 mg/dL. Magnesium was within normal limits. Measured serum osmolality was low at 223 mmol/kg, which is the same as calculated serum osmolality. Urine sodium was 58, and urine osmolality was 613. Thyroid-stimulating hormone (TSH) was elevated at 133 uIU/mL (reference range: 0.35-5.50 uIU/mL), free T4 was low at 0.16 ng/dL (reference range: 0.76-1.46 ng/dL), free T3 was low at <0.6 pg/mL (reference range: 2.2-4.2 pg/mL). She was given a one-time dose of levothyroxine at 300 mcg, then followed by 100 mcg daily.

**Figure 1:**
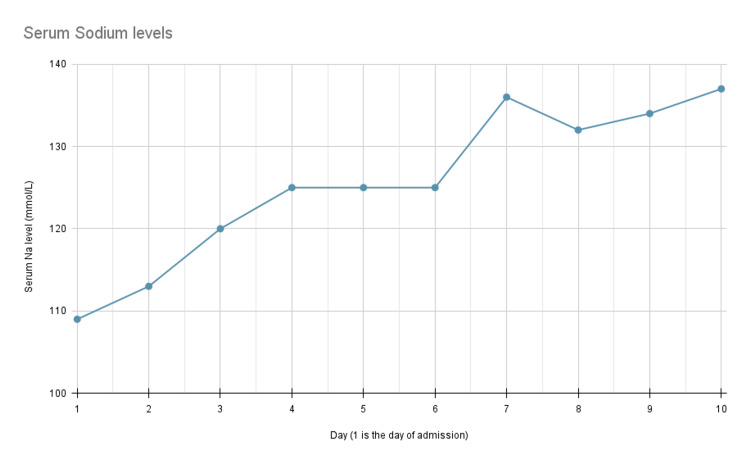
Serum sodium levels This figure depicts the hospital day on the x-axis and serum Na on the y-axis. Na levels in this figure were obtained in the morning, except on day one, which correlates with the day of admission.

During this admission, she was initially treated for a presumed SIADH with 3% hypertonic saline at 10 mL/hr. Serum sodium was checked frequently to ensure safe and adequate correction (Figure 1). Electrolytes abnormalities were addressed appropriately. On day two, the infusion rate was increased to 20 mL/hr and then increased again to 30 mL/hr on day five. On day three of the patient's hospital stay, serum adrenocorticotropic hormone (ACTH) was measured and found to be less than 2 pg/mL (reference range: 7-63 pg/mL), and random serum cortisol was found to be 7.7 ug/dL. Critically ill patients with a random cortisol level lower than 25 ug/dL may suggest adrenal insufficiency [8]. 

Given the history of melanoma, magnetic resonance imaging (MRI) of the brain with and without contrast was obtained and found to be negative for metastatic disease. At the same time, medical records from her oncologist were obtained, which revealed that she was treated with pembrolizumab and had developed hypothyroidism and suspected adrenal insufficiency (due to hypotension and hyponatremia). The oncologist prescribed prednisone 20 mg daily with plans for the patient to follow up with an endocrinologist; however, she reported non-adherence. Given hypotension and hyponatremia, an echocardiogram was obtained by the oncologist as well and revealed a left ventricle ejection fraction of 70 percent, and grossly unremarkable. 

On day seven, she was started on hydrocortisone 100 mg three times daily, and the hypertonic saline infusion was discontinued. Sodium levels became stable without hypertonic saline infusion (Figure 1). 

## Discussion

We would like to discuss, briefly, the most likely cause of hyponatremia in this patient. Hypothyroidism and adrenal insufficiency have been reported to be associated with hyponatremia. Patients with severe hypothyroidism leading to myxedema coma and reduced cardiac output may develop hyponatremia. Hypothyroidism is not direct causation of hyponatremia. Hypothyroidism decreases cardiac output and subsequently increases plasma antidiuretic hormone (ADH) concentration and water retention, precipitating hyponatremia [9].

Adrenal insufficiency can be classified as primary, secondary, or tertiary. Primary adrenal insufficiency is caused by adrenocortical failure [10]. Secondary adrenal insufficiency develops from impairment of the pituitary gland, resulting in low ACTH secretion. Pembrolizumab-induced hypophysitis occurs in <1% of the patients [10]. Tertiary adrenal insufficiency results from impairment in the hypothalamus. 

**Figure 2:**
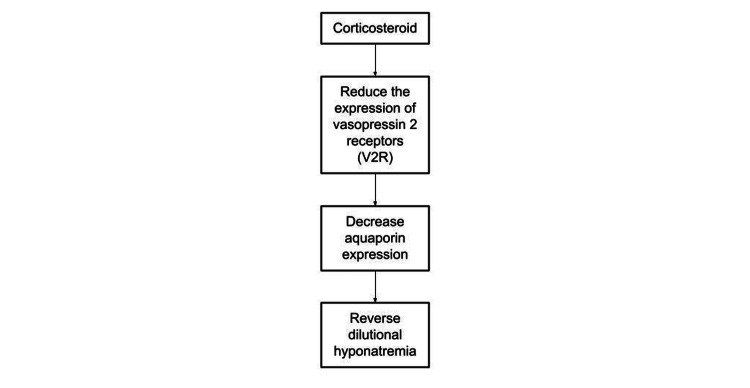
Cortisol induces reverse dilutional hyponatremia in rats

We suspected a secondary adrenal insufficiency given her low ACTH level (less than two) and inappropriately normal cortisol level during shock. She was started on corticosteroid. Cortisol deficiency impairs renal excretion of free water and increases the plasma level of antidiuretic hormone, vasopressin (AVP) [11,12]. In a study conducted on rats, corticosteroids were found to reduce the expression of vasopressin 2 receptors (V2R), decrease aquaporin expression, and subsequently reverse dilutional hyponatremia (Figure 2) [13]. 

## Conclusions

Pembrolizumab is an anti-PD-1 ICI approved for various malignancies with multiple adverse endocrine-related effects, including hypothyroidism and secondary adrenal insufficiency. In this case report, we present a patient who developed hypothyroidism and secondary adrenal insufficiency and presented with severe hyponatremia. Both conditions may precipitate hyponatremia; however, the hypothyroidism mechanism is secondary to reduced cardiac output. The patient did not have clinical or imaging evidence of decreased cardiac output. We believe that her hyponatremia was mainly secondary to adrenal insufficiency.
